# Multidisciplinary palliation for unresectable recurrent rectal cancer: hypoxic pelvic perfusion with mitomycin C and oxaliplatin in patients progressing after systemic chemotherapy and radiotherapy, a retrospective cohort study

**DOI:** 10.18632/oncotarget.26972

**Published:** 2019-06-11

**Authors:** Stefano Guadagni, Giammaria Fiorentini, Andrea Mambrini, Francesco Masedu, Marco Valenti, Andrew Reay Mackay, Donatella Sarti, Enrico Ricevuto, Marco Clementi, Marco Catarci, Gianni Lazzarin, Gemma Bruera

**Affiliations:** ^1^ Department of Applied Clinical Sciences and Biotechnology, University of L’Aquila, L’Aquila, Italy; ^2^ Department of Oncology and Hematology, Azienda Ospedaliera “Ospedali Riuniti Marche Nord”, Pesaro, Italy; ^3^ Oncology Unit, Azienda USL Toscana Nord Ovest, Massa Carrara, Italy; ^4^ Oncology Territorial Care S. Salvatore Hospital, Oncology Network ASL1 Abruzzo, L’Aquila, Italy; ^5^ General Surgery Unit, “C. e G. Mazzoni” Hospital, Ascoli Piceno, Italy

**Keywords:** hypoxic pelvic perfusion with mitomycin C and oxaliplatin, unresectable recurrent rectal cancer, retrospective cohort study

## Abstract

**Background:**

Innovative systemic treatments and loco-regional chemotherapy by hypoxic pelvic perfusion (HPP) have been proposed for unresectable recurrent rectal cancer (URRC). Regorafenib and trifluridine-tipiracil reported significantly increased PFS 1.9-2.0 months, OS 6.4-7.1 months vs placebo, respectively. Present study evaluated safety and efficacy of mitomycin/oxaliplatin HPP associated to intravenous cetuximab, and of third line systemic therapy in clinical practice.

**Methods:**

HPP consisted of: isolation, perfusion, chemofiltration. Patients received mitomycin 25 mg/m^2^ and oxaliplatin 80 mg/m^2^ during HPP; from days 21 to 28, cetuximab 250 mg/m^2^/week. In case of partial response or stable disease, HPPs were repeated every 8 weeks. In control group, systemic third and further lines of therapy were defined in clinical practice according to clinical (age, comorbidities, performance status), biological parameters (*KRAS*, *NRAS*, *BRAF* genotype).

**Results:**

From 2005 to 2018, 49 URRC patients were enrolled; 33 in HPP/target-therapy, 16 in systemic therapy control group. No HPP related complications were reported. Most common adverse events were skin, bone marrow toxicities. In HPP/target-therapy group, ORR and DCR were 36.4 and 100%; in systemic therapy control group, 18.7 and 31.25%, respectively. In HPP/target-therapy compared with systemic therapy group, respectively, DCR seemed significantly favourable (*P* = 0.001), as PFS 8 vs 4 months (*P* = 0.018), and OS 15 vs 8 months (*P *= 0.044).

**Conclusions:**

Present data showed that integration of HPP/target-therapy may be effective in local control, and efficacy as third line treatment of URCC patients. This therapeutic strategy deserves further prospective randomized trials to be compared to conventional systemic treatments.

## INTRODUCTION

The incidence of local recurrence in rectal cancer has decreased to approximately 8% in the last years [1]. Unfortunately, 50% of recurrent cancers are evaluated as unresectable in high volume specialist centers [2]. For unresectable recurrent rectal cancer (URRC) patients in progression after systemic chemotherapy and radiation, several palliative therapies have been proposed [3–5]. In real life experience of metastatic colorectal cancer (mCRC) patients progressing after an intensive first line FIr-B/FOx [6–8], or FIr-C/FOx-C [9], treatment in fit patients, or conventional medical treatment regimens in unfit patients [10, 11], we reported a median progression-free survival (PFS) of 10 months and an overall survival (OS) of 14 months. Recently, third line treatments with the multitargeted tyrosine kinase inhibitor regorafenib [12, 13], and the cytotoxic drug trifluridine/tipiracil (TAS-102), consisting of the association between trifluorothymidine, and thymidine-phosphorilase-tipiracil, that inhibits trifluorothymidine degradation [14], of mCRC patients showed increased efficacy compared with placebo controls in randomized studies. Regorafenib addition to best supportive care increased OS in mCRC patients who have received all approved standard therapies [12]: median PFS 1.9 vs 1.7 months, median OS 6.4 vs 5.0 months, in the regorafenib compared to placebo arm. In the Recourse trial [14], evaluating TAS-102, all the patients had received prior chemotherapy regimens containing fluoropyrimidine, oxaliplatin, and irinotecan, all but one patient had received bevacizumab, all but two patients with KRAS wild-type tumors had received cetuximab or panitumumab; median PFS was 2.0 vs 1.7 months, median OS was 7.1 vs 5.3 months, in the TAS-102 compared with placebo group, respectively.

Few specialists centers performed palliative loco-regional chemotherapy by hypoxic pelvic perfusion (HPP) [15]. HPP is a reasonably complex procedure that integrates the multidisciplinary competence of surgeons, radiologists and perfusionists, and involves isolation of the pelvic circulation by blocking blood flow in the aorta and inferior vena cava with balloon catheters, and at thigh-level with pneumatic cuffs [15]. The rationale of this loco-regional chemotherapeutic approach is based upon the possibility to expose tumors to high drug concentrations within the perfused compartment, and the use of chemotherapeutic agents, such as mitomycin C, that exhibit enhanced toxicity under conditions of hypoxia [16, 17]. Pharmacokinetic studies demonstrated that HPP can be interchangeably performed by either surgical [18], or percutaneous approaches [19, 20]. With respect to efficacy, median survival times from initial HPP range between 10 to 20 months in non-homogeneous studies in pretreated patients with HPP-procedures associated to mono- or combination chemotherapy [21], single or repetitive treatments and high (cisplatin 170 mg/m^2^, 5-fluorouracil 1000 mg/m^2^) [22] or low drug mitomycin C (25 mg/m^2^) dosages [23]. We recently reported that HPP associated to mitomycin C (25 mg/m^2^) in patients with URRC in progression after systemic chemotherapy and radiotherapy, provided tumor response rate 40%, median PFS 6 months, and median OS 10 months [23].

The purpose of this retrospective cohort study of URRC patients in progression after two lines of systemic chemotherapy and radiotherapy was to evaluate safety and efficacy of a cohort of patients treated with mitomycin/oxaliplatin HPP associated to intravenous cetuximab, and a control cohort of patients treated with third and further lines of systemic therapy. This control cohort was based on URRC patients progressing to second line chemotherapy included in our previously reported real life experience of mCRC patients [6–11].

## RESULTS

### Patients characteristics

Over the years from 2005 to 2018, 49 patients with unresectable recurrent rectal cancer (URRC), progressing after two lines of systemic chemotherapy and radiotherapy were enrolled; 33 patients were treated in HPP/target-therapy cohort (mitomycin/oxaliplatin HPP associated to intravenous cetuximab) and 16 patients in the systemic therapy control group (all conventional systemic treatments according to patients fitness, age, performance status (PS), and comorbidity status). A cross-sectional study identified patient demographic and baseline data, displayed in Table 1. Recurrences were subdivided in three groups using a modified Yamada’s classification [24]: localized (including cases with invasion of uterus, vagina, bladder, prostate, and seminal vesicles), sacral, and lateral. Based on Eastern Cooperative Oncology Group (ECOG) classification (approximately 80% ECOG 3) and symptoms as pain, tiredness, and lack of appetite, the clinical profile of severity burden was between moderate and severe for all 49 patients [25].

**Table 1:** Characteristics of the 49 URRC patients submitted to HPP/target-therapy or systemic therapy

	All patients (n=49)	HPP/target-therapy group (n=33)	Systemic therapy group (n=16)	*P* value	Test
**Gender**				0.437 (ns)	Student’t
- Male	33	21	12		
- Female	16	12	4		
**Age** (years, median/iqr) **at the 1^st^ treatment of the 3^rd^ line**	61/56-68	60/56-65	67.5/57-69	0.108 (ns)	Mann-Whitney
**Previous treatments of primary tumor**					
- neo-adjuvant chemo/RT	6	4	2	0.970 (ns)	Chi square
- abdominoperineal resection	28	19	9	0.930 (ns)	Chi square
- low anterior resection	21	14	7	0.930 (ns)	Chi square
- adjuvant chemo/RT	18	12	6	0.938 (ns)	Chi square
**Previous treatments of recurrence**					
- Systemic therapy	49	33	16	Not applicable	
--chemotherapy	49	33	16	Not applicable	
---Fluorouracil	48	33	15	0.327 (ns)	Fisher exact
---Oxaliplatin	48	33	15	0.327 (ns)	Fisher exact
---Irinotecan	49	33	16	Not applicable	
---Capecitabine	7	5	2	1.000 (ns)	Fisher exact
--targeted-therapy	45	30	15	1.000 (ns)	Fisher exact
--cetuximab	12	8	4	1.000 (ns)	Fisher exact
--bevacizumab	44	29	15	1.000 (ns)	Fisher exact
- RT	12	9	3	0.515 (ns)	Chi square
- Surgery	11	10	1	0.076 (ns)	Fisher exact
**Yamada’s modified classification^20^**					
- localized (*)	13	9	4	0.891 (ns)	Student’t
- sacral	27	18	9		
- lateral	9	6	3		
**Other metastatic sites**					
- not	22	16	6	0.479 (ns)	Student’t
- yes	27	17	10		
**Eastern Cooperative Oncology Group (ECOG)**					
- 3	39	26	13	0.845 (ns)	Student’t
- 2	10	7	3		
**Interval time from URRC diagnosis and the 1^st^ treatment of the 3^rd^ line **(months, median/iqr)	13/10-16	13/10-16	14/10.5-30	0.211 (ns)	Mann-Whitney
**Number of cycles of the 3^rd^ line** (mean/SD)	2.61/1.40	2.36/1.50	3.12/1.02	0.07 (ns)	Student’t
**Samples evaluated for^#^**				Not applicable	
**KRAS genotype**	19	3	16		
wild-type	12	3	9		
mutant	8	-	7		
**NRAS genotype**	3	3	-		
**BRAF genotype**	6	-	6		
wild-type	6	-	6		
mutant	-	-	-		

Chemo/RT = systemic chemotherapy/radiotherapy; iqr = interquartile range; SD = standard deviation; (^*^) this group included cases with invasion of uterus, vagina, bladder, prostate, seminal vesicles; URRC = unresectable recurrent rectal cancer; ^#^anti-EGFR drugs administered according to racommendations alongtime (evaluation of EGFR overexpression, KRAS exon 2 wild-type, KRAS/NRAS exon 2-4 wild-type).

### Adverse events

No technical, hemodynamic or vascular complications were detected during HPP procedures and vascular cannulation was possible in all cases. There were no treatment-related deaths. Procedure-related complications and toxicities are listed in Table 2. The most common treatment-related adverse reactions were skin toxicity and bone marrow hypocellularity. No grade 4 hematological toxicity was observed in either group and no serious myelosuppression occurred. No significant differences were detected between groups using not-parametric tests.

**Table 2:** Procedure-related complications and toxicities in 49 URRC patients submitted to HPP/target-therapy or systemic therapy

**Part A: Procedure-related complications**	**Grade**	**All patients (n=49)**	**HPP/target-therapy group (n=33)**	**Systemic therapy group (n=16)**
Persistent leakage of fluid from the incision	2	1	1	0
Seroma	1	1	1	0
Wound infection	1	1	1	0
Scrotum edema	1	1	1	0
Pelvic pain	1	2	1	1
Inguinal hematoma	1	1	1	0
Port-a-cath infection	2	1	0	1
**Part B: Procedure-related complications**	**Grade**	**All patients (n=49)**	**HPP/target-therapy group (n=33)**	**Systemic therapy group (n=16)**
Bone marrow hypocellularity	1	8	4	4
	2	0	0	0
	3	4	2	2
Platinum-induced neurotoxicity	2	4	2	2
Alopecia	2	2	2	0
Nausea and vomiting	1	4	3	1
Diarrhea	1	2	0	2
	2	4	0	4
Mucositis	3	1	0	1
Fatigue	1	5	2	5
	2	2		0
Skin toxicity	1	6	4	2
	2	18	12	6
	3	4	3	1

### Tumor response

Table 3 shows the results of tumor response evaluated according to RECIST 1.1, considering the two first treatments of the third line for both groups. Among the 33 patients in the HPP/target-therapy group, twelve (36.4%) partial responses (PRs), and twenty-one (63.6%) stable diseases (SDs) were observed; the objective response rate (ORR) and disease control rate (DCR) were 36.4% and 100%, respectively. Among the 16 patients in the systemic therapy control group, three (18.7%) PRs, two (12.5%) SDs, and eleven (68.7%) progressive diseases (PDs) were observed; the ORR and DCR were 18.7 and 31.25%, respectively. The HPP/target-therapy group provided a significantly higher DCR in comparison to the systemic therapy control group (P = 0.001).

**Table 3:** Comparison of response between the HPP/target-therapy group and Systemic therapy control group in 49 URRC patients

Outcome	HPP/target-therapy group (n=33)	Systemic therapy group (n=16)	χ^2^	*P* value
Partial response (PR)	12 (36.36%)	3 (18.75%)	29.782	0.001
Stable disease (SD)	21 (63.64%)	2 (12.50%)		
Progressive disease (PD)	0 (0.00%)	11 (68.75%)		
Objective response rate (ORR)	12 (36.4%)	3 (18.7%)	1.573	0.210 (ns)
Disease control rate (DCR)	33 (100%)	5 (31.2%)	29.255	0.001

Among 26 PS3 patients treated with HPP, an improvement to PS2 was reported in 6 patients after treatment (23%).

### Survival

**Figure 1:**
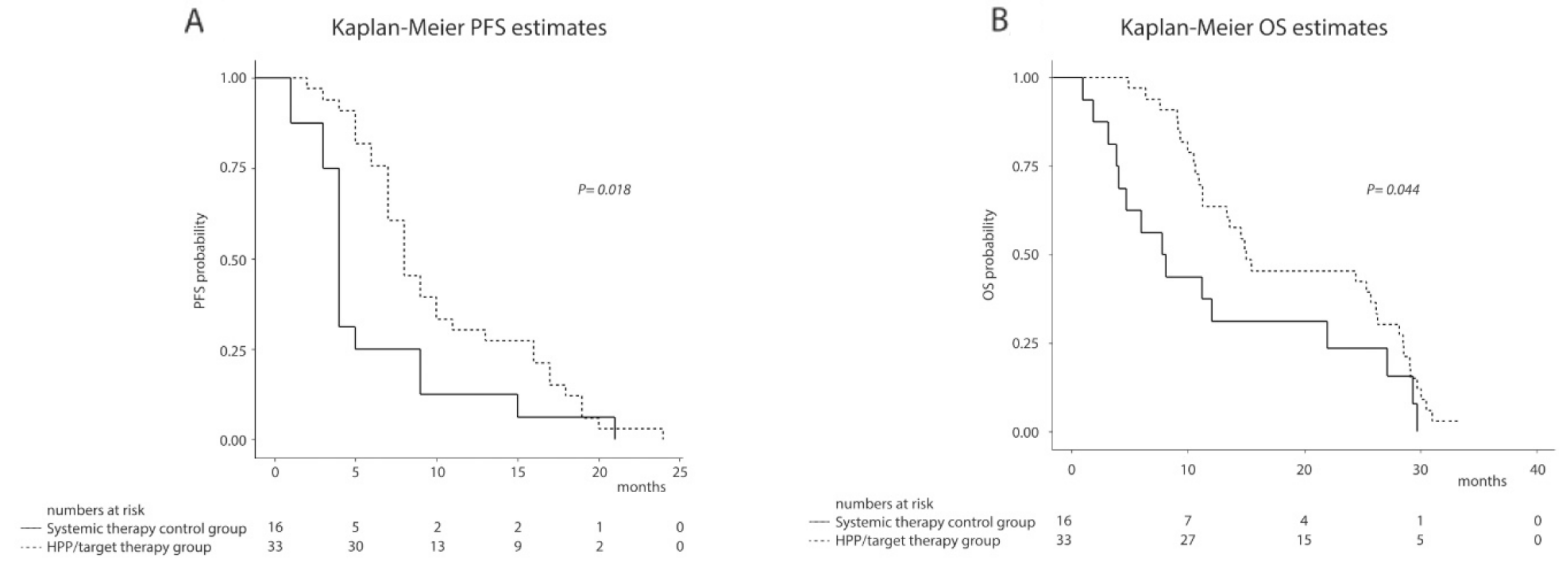
Kaplan-Meier survival estimates in 49 URRC patients from first treatment of the third line to end of follow-up: **(A)** Progression free survival; **(B)** Overall survival.

The median follow-up time was 14 (iqr 9 - 27) months. At the end of the follow-up period, 1 (3%) patient in the HPP/target therapy group and 1 (6.0%) patients in the systemic therapy control group were still alive. A Kaplan-Meier survival analysis showed significant differences between the two groups in terms of PFS and OS. The median PFS (from first treatment of the third line) was 8 (iqr 7 - 16) months in the HPP/target therapy group and 4 (iqr 3.5 - 7) months in the systemic therapy control group, respectively (P = 0.018) (Figure 1A). The median OS (from first treatment of the third line) was 15 (iqr 11 - 28) months in the HPP/target therapy group and 8 (iqr 4 - 19.5) months in the systemic therapy control group (P = 0.044) (Figure 1B).

The Cox univariate and multivariate analysis identified several prognostic factors of PFS and OS (Table 4). The univariate analysis demonstrated that the third line treatment modality, modified Yamada’s classification [24], and ECOG displayed a significant association with PFS (Table 4-Part A). Of these, the third line treatment modality (HR = 2.306, 95% CI 1.187 - 4.479, P = 0.014), and modified Yamada’s classification [24] (HR = 6.158, 95% CI 2.321 - 16.337, P = 0.001) were further confirmed by multivariate analysis to be independent predictive factors for PFS. The univariate analysis demonstrated that the third line treatment modality, gender, age, modified Yamada’s classification [24], and ECOG displayed an association with OS (P < 0.10). Of these, the modality of third line treatment (HR = 6.175, 95% CI 2.726 - 13.989, P = 0.001), age ≤ 60 years (HR = 2.282, 95% CI 1.089 - 4.779, P = 0.029), type sacral versus localized of modified Yamada’s classification (HR = 2.749, 95% CI 1.214 - 6.225, P = 0.015), type lateral versus localized of modified Yamada’s classification (HR = 15.809, 95% CI 4.813 – 51.928, P = 0.001), and ECOG (HR = 22.637, 95% CI 5.844 - 87.681, P = 0.001) were further confirmed by multivariate analysis to be independent predictive factors for OS.

**Table 4:** Part A: Progression free survival (PFS) times from first treatment of the third line to death or last contact in 49 URRC patients in progression after two lines systemic chemotherapy and radiotherapy. Patients were stratified according to third line treatment modality, gender, age, Yamada’s modified classification, other sites of metastases, and ECOG. Part B: Survival times

**Part A-Progression free survival**					
**Variables (number of patients)**	**Median (months)/iqr**	**Log-Rank χ^2^**	***P* value**	**HR (95% CI)**	***P* value**
Third line treatment					
- Systemic therapy control group (n = 16)	4/3.5 - 7			1.984 (1.071-3.673)	0.029
- HPP/target therapy group (n = 33)	8/7 - 16	5.52	0.019		
Gender					
- Male (n = 33)	6/4 - 10			1.156 (0.625 - 2.137)	0.644 (ns)
- Female (n = 16)	9/7 - 14	0.24	0.622 (ns)		
Age					
- ≤60 (n = 24)	7/5 - 10.5			0.918 (0.519 - 1.623)	0.769 (ns)
- >60 (n = 25)	8/4 - 13	0.10	0.755 (ns)		
Yamada’ s modified classification^20^					
- localized* (n = 13)	9/7 - 17				
- sacral (n = 27)	7/4 - 15			1.277 (0.654 - 2.494)	0.473 (ns)
- lateral (n = 9)	3/2 - 5	13.41	0.001	4.069 (1.669 - 9.917)	0.002
Other sites of metastases					
- Yes (n = 27)	7/4 - 10			1.197 (0.676 - 2.116)	0.537 (ns)
- Not (n = 22)	8/5 - 16	0.43	0.512		
ECOG					
- 3 (n = 39)	7/4 - 10			2.073 (0.987 - 4.352)	0.054
- 2 (n = 10)	9/8 - 19	4.34	0.037		
**Part B – Survival**					
**Variables (number of patients)**	**Median (months)/iqr**	**Log-Rank χ^2^**	***P* value**	**HR (95% CI)**	***P* value**
Third line treatment					
- Systemic therapy control group (n = 16)	8/4 - 19.5			1.799 (0.969 - 3.343)	0.063
- HPP/target therapy group (n = 33)	15/11 - 28	4.05	0.044		
Gender					
- Male (n = 33)	11/8 - 26			1.678 (0.905 - 3.112)	0.100 (ns)
- Female (n = 16)	24.5/14 - 29	3.19	0.074		
Age					
- ≤60 (n = 24)	13.5/9 - 24.5			0.616 (0.339 - 1.118)	0.111 (ns)
- >60 (n = 25)	15/8 - 29	2.92	0.088		
Yamada’ s modified classification^20^					
- localized* (n = 13)	27/22 - 29				0.003
- sacral (n = 27)	12/8 - 26			1.605 (0.816 - 3.157)	
- lateral (n = 9)	6/3 - 11	10.68	0.005	3.765 (1.566 - 9.049)	
Other sites of metastases					
- Yes (n = 27)	11/6 - 15			1.532 (0.855 - 2.743)	0.151
- Not (n = 22)	25.5/15 - 29	2.41	0.120		
ECOG					
- 3 (n = 39)	11/8 - 17			11.917 (3.857 - 36.817)	0.001
- 2 (n = 10)	30/29 - 30	25.57	0.001		

Median overall survival from recurrent rectal cancer diagnosis to death or end of follow-up (RRC-OS) was 30 (iqr 21 - 42) months.

Among HPP procedure group, patients did not underwent subsequent lines of treatment. Among patients treated with systemic treatments, 5 underwent further lines (30%).

## DISCUSSION

The management of URRC requires a multidisciplinary approach and when standard treatments such as systemic chemotherapy and radiotherapy failed or are impracticable, the combination of a locoregional chemotherapy modality, as HPP, and systemic therapy may be an alternative option and it is under investigation [19]. This is the first paper evaluating combination of locoregional chemotherapy and systemic therapy versus a historical control of systemic therapy as third line for URRC, defined in clinical practice according to clinical (age, comorbidities, performance status), biological parameters (KRAS, NRAS, BRAF genotype).

The historical control of URRC patients, progressing after second line treatments and included in our previously reported real life experience of overall pre-treated mCRC patients, showed a median OS of 4 months, consisting with the median OS of approximately 5 months for the control arms in recently reported phase III trials indicating regorafenib or TAS-102 as innovative third line treatments [13, 14].

The present study showed that the DCR of the HPP/target-therapy group (mitomycin, oxaliplatin and cetuximab) seems non inferior but also potentially significantly higher (P = 0.001) than the systemic therapy control group, suggesting that the locoregional and systemic combination regimen may be beneficial for the short-term control of URRC lesions. Patients treated with HPP approach were prevalently PS3, approximately 80%, due to disease related symptoms burden, thus suggesting the potential benefits of this treatment also in terms of palliative care. The analysis of efficacy seems to show that median PFS (from the first third line treatment) of the HPP/target-therapy group and the systemic therapy control group were 8 months and 4 months, respectively, with statistically significant difference between them (P = 0.018); median OS (from the first third line treatment) of the HPP/target-therapy group and the systemic therapy control group were 15 and 8 months, respectively, with statistically significant difference between them (P = 0.044); the above results shows that URRC patients may benefit from combination of locoregional chemotherapy and systemic therapy. The multivariate analysis upon the 49 URRC patients evaluated in this study showed that the locoregional and systemic combination of treatment, age > 60 years, localized type of recurrence, and ECOG 2 class may be independent predictors of prolonged OS. The pharmacokinetic and microenvironmental advantages of HPP associated to target-therapy, in terms of tumor drug exposure and enhanced activity under hypoxic condition for specific chemotherapeutic agents, provide a better control of localized rectal cancer recurrence in comparison to systemic therapy alone [26]. The present study demonstrated that the combination of locoregional and systemic therapy, as in other cancers [27], may provide benefit also in URRC patients with other metastatic sites.

Common adverse events were manifested as skin toxicity and bone marrow hypocellularity and there was no statistically significant difference between the two groups, indicating that URRC patients are tolerant of the two regimens and that HPP is, in general, safe and reliable, even with more limitations regarding different administered chemotherapy regimens. With respect to the tolerability of locoregional chemotherapy in association with chemofiltration, we confirm similar reports [22, 28], with absence of nephrotoxicity, severe neuropathy, and G4 hematological toxicity.

There were several limitations in the study: i) this study was conducted in a single center for homogeneity of HPP technique; ii) the study is retrospective and not prospective; iii) the number of cases is relatively low insufficient, and the results may be biased.

With limitations concerning retrospective evaluation and statistical relevance of OS results, present data show that in principle integration of HPP/target-therapy may be effective at symptomatic level in the local control, and in terms of long-term efficacy of URCC patients. In conclusion, HPP with mitomycin and oxaliplatin associated to cetuximab target-therapy is an efficient and safe alternative choice for third line treatment of URCC patients which deserves further prospective randomized trials.

## MATERIALS AND METHODS

### Patient population

This retrospective cohort study of patients with recurrent rectal cancer was enrolled at the University of L'Aquila, L'Aquila, Italy, after approval by the investigational review board [Ethics committee of “ASL n.1, Abruzzo, Italy; Chairperson: G. Piccioli; protocol number 10/CE/2018; date of approval: 19 July, 2018 (n.1419)], providing that all patients had unresectable disease. All patients received complete information about their disease and the implications of the proposed palliative treatment, in accordance with both Declaration of Helsinki and ethical standards of the committee on human experimentation at our institution, and written consent was obtained.

Patient eligibility criteria were: (i) histological diagnosis of adenocarcinoma of the rectum; (ii) diagnosis of unresectable recurrent rectal cancer, defined by pelvic side-wall involvement, and/or growth into the sciatic notch, and/or involvement of the first and/or second sacral vertebra, and/or encasement of the bladder or iliac vessels; (iii) an increase in recurrent tumor size for at least three months following systemic chemotherapy or radiation; (iv) a performance status of 0-3 based upon the Eastern Cooperative Oncology Group (ECOG) scale; (v) a leukocyte count > 2500 cells/mm^3^ and platelet count > 50000 cells/mm^3^; (vi) a serum Creatinine concentration of ≤ 1.2 mg/dL; (vii) absence of liver failure, deep venous thrombosis, severe atherosclerosis, or coagulopathy; (viii) URRC patients in progression after two lines of systemic chemotherapy.

According to recommendations for administration of anti-epidermal growth factor receptor (anti-EGFR) drugs, tumoral samples of primary tumors or metastatic site were evaluated for EGFR expression, and mutations in KRAS (Kirsten rat sarcoma virus), NRAS (neuroblastoma RAS viral oncogene homolog) exon 2 codons 12 and 13, exon 3 codons 59 and 61 and, exon 4 codons 117 and 146, and BRAF genes.

### HPP techniques

**Figure 2:**
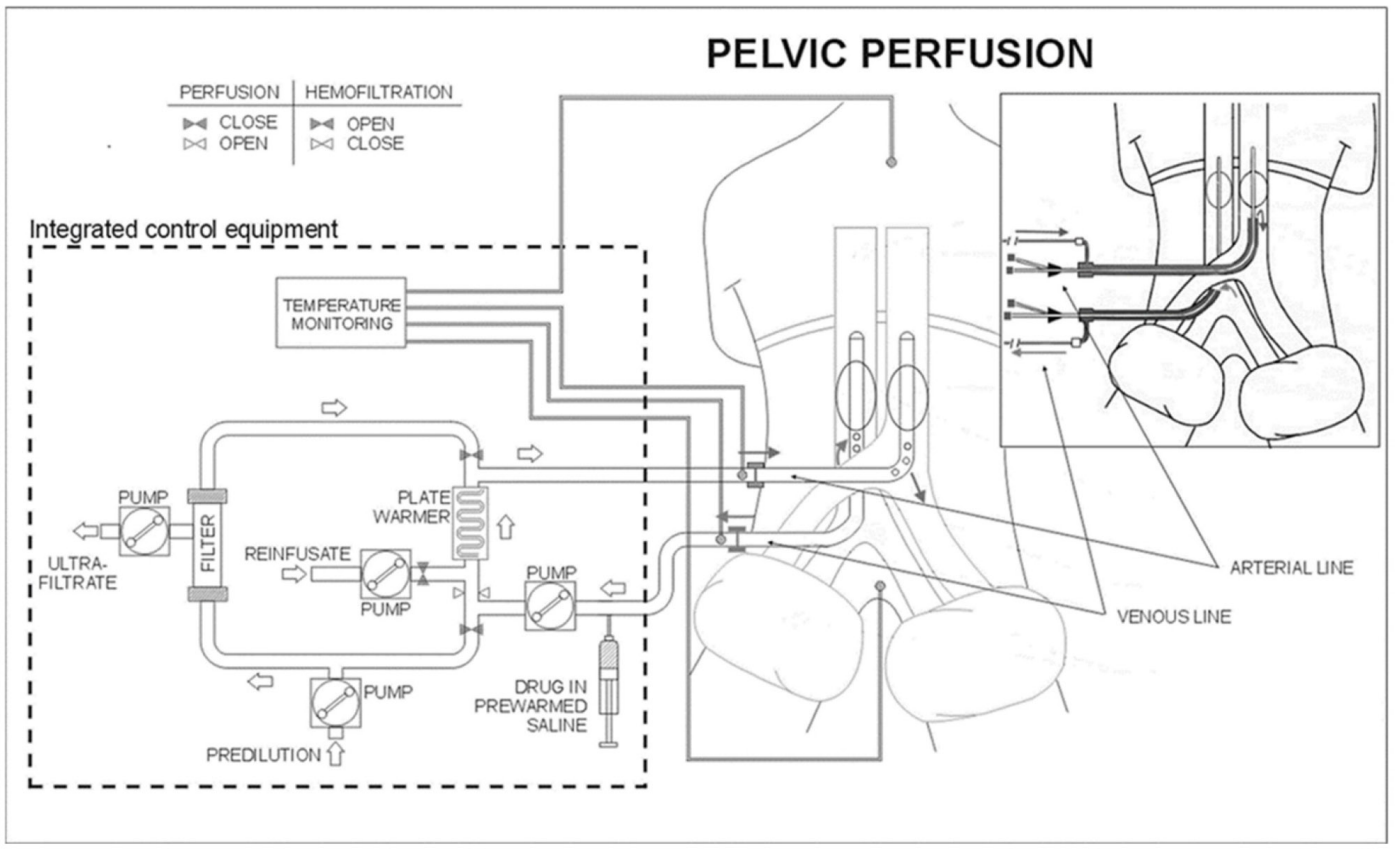
Schematic representation of the surgical and percutaneous hypoxic pelvic perfusion (HPP) procedures, with chemofiltration

Prior to perfusion, patients were subjected to aortoiliac tree and inferior vena cava angiography or angio-computerized tomography (CT). Surgical or percutaneous perfusions were performed under general anesthesia. In patients exhibiting femoral vessel fibrosis, requiring 2 or 3 repeat perfusions, the surgical approach was achieved by exposing the iliac vessels. Percutaneous perfusion was not performed if the common femoral artery diameter was ≤7 mm, making vessel dissection risky. The surgical approach was preferred for all patients and the percutaneous approach was reserved for patients submitted to more than 3 perfusions. HPP (Figure 2) comprised three phases: isolation, perfusion and chemofiltration, as previously described [19].

### Drugs regimens

HPP/target therapy group schedule: at HPP, patients received mitomycin (Mitomycin C, Kyowa Kirin, Milan, Italy), at the dose of 25 mg/m^2^ based on our previous studies [18, 19], and oxaliplatin (Eloxatin; Sanofi-Aventis, Milan, Italy), at the dose of 80 mg/m^2^ according to literature reports on locoregional chemotherapy palliation of metastatic colorectal cancer patients [29]. Starting from days 21 to 28 after HPP, if the local cutaneous toxicity was ≤ grade 1, patients received cetuximab (Erbitux; Merck, Darmstadt, Germany), at the dose of 250 mg/m^2^, intravenous infusion in NaCl 0.9% 600 ml over 120 minutes, days 1, 8, 15, 22. In patients exhibiting a partial response or stable disease, HPPs were repeated at intervals of approximately 8 weeks based upon a pilot study [18], in which progression was always observed in presence of residual tumor. Treatment was not repeated in patients exhibiting a complete response; if local recurrence or distant relapse had progressed > 20% in dimension; if simultaneous distant relapses occurred; if the general condition of the patient worsened or if the patient did not consent.

Systemic therapy control group schedules, defined according to clinical (age, comorbidities, performance status), and biological parameters (KRAS, NRAS, BRAF genotype): oxaliplatin (Eloxatin; Sanofi-Aventis, Milan, Italy) as 2-hours intravenous infusion in dextrose 5% 250 ml, 70-80 mg/m^2^, days 1, 15 [30], or irinotecan (Campto; Pfizer, Latina, Italy) 120-160 mg/m^2^, as 90 minutes intravenous infusion in NaCl 0.9% 250 ml, days 1, 15, added to cetuximab (Erbitux; Merck, Darmstadt, Germany), loading dose 400 mg/m^2^, followed by 250 mg/m^2^, intravenous infusion in NaCl 0.9% 600 ml over 120 minutes at first time, then in 300 ml over 60 minutes, days 1, 8, 15, 22, every 28 days; panitimumab (Vectibix; Amgen, Breda, Netherlands) 6 mg/kg, as 90 minutes intravenous infusion at first administration, 60 minutes at subsequent ones, in NaCl 0.9% 100 ml, days 1, 15, every 28 days; panitumumab added to irinotecan according to the previously reported administration modality; oxaliplatin added to timed-flat infusion 5-fluorouracil (Fluorouracil Teva; Teva Italia, Milan, Italy) 750-900 mg/m^2^/day, over 12-hour (from 10:00 p.m to 10:00 a.m.), days 1-2, 8-9, 15-16, 22-23; 5-fluorouracil added to bevacizumab (Avastin®, Roche), 5 mg/kg, administered over 90 minutes at the first, 60 minutes at the second and 30 minutes from the third time, intravenous infusion in 100 ml of NaCl 0.9%, days 1 and 15, every 28 days; raltitrexed (Tomudex, Hospira, Naples, Italy) 3 mg/m^2^, as 15 minutes intravenous infusion in NaCl 0.9% 250 ml, days 1, every 21 days; capecitabine (Xeloda, Roche, Grenzach-Wyhlen, Germay) 825 mg/m^2^/twice a day orally administered, days 1-14, every 21 days; regorafenib (Stivarga, Bayer, Leverkusen, Germany) 80-160 mg/day, days 1-21, every 28 days. Treatment was discontinued in case of progressive disease, worsening of general conditions, severe adverse events, or patient withdrawal. In both groups, cetuximab and panitumumab were administered according to the following conditions: EGFR overexpression; absence of mutations in KRAS and NRAS exon 2 codons 12 and 13, exon 3 codons 59 and 61 and, and exon 4 codons 117 and 146, in recurrent cancer cells or primary tissues [31].

### Criteria for responses and adverse events

Tumor responses were assessed in accordance with Response Evaluation Criteria in Solid Tumors (RECIST version 1.1), at 30-45 days following each treatment [32]. The responses of patients treated prior to 2009, were re-classified retrospectively. Responses were evaluated by CT, Magnetic Resonance Imaging (MRI), and Position-emission Tomography (PET). Adverse events were evaluated in accordance with the Common Terminology Criteria for Adverse Events of the National Cancer Institute (CTCAE v4.03).

### Statistical analysis

Statistical analyses were performed using STATA software, version 14 (StataCorp, College Station, Texas). Statistics were calculated with 95% confidence limits. Statistical analyses were performed using t tests or Mann-Whitney tests for measurement data, and Chi-square tests or Fisher’s exact tests for count data. Survival-rates were estimated using the Kaplan-Meier product limit estimator and no patients were lost during follow-up. Survival times were stratified according to clinical variables that may affect survival and log-rank tests were used to assess significant differences between groups. Hazard ratios were estimated using a proportional hazard Cox regression model. For both groups, progression-free survival-time (PFS) and overall survival (OS) were calculated from the first treatment of the third line to death or end of follow-up. RRC overall survival (RRC-OS) was calculated from diagnosis of RRC to death or end of follow-up.
